# Benefits Derived from Full Laboratory Automation in Microbiology: a Tale of Four Laboratories

**DOI:** 10.1128/JCM.01969-20

**Published:** 2021-02-18

**Authors:** Karissa Culbreath, Heather Piwonka, John Korver, Mir Noorbakhsh

**Affiliations:** a Department of Infectious Disease, TriCore Laboratories, Albuquerque, New Mexico, USA; b Department of Microbiology, Willis-Knighton Health Systems, Shreveport, Louisiana, USA; c Department of Microbiology, Hamilton Regional Medicine Laboratory Program, Hamilton, Ontario, Canada; d Department of Microbiology, Sutter Health Shared Laboratory, Livermore, California, USA; NorthShore University HealthSystem

**Keywords:** efficiency, laboratory automation

## Abstract

Automation in clinical microbiology is starting to become more commonplace and reportedly offers several advantages over the manual laboratory. Most studies have reported on the rapid turnaround times for culture results, including times for identification of pathogens and their respective antimicrobial susceptibilities, but few have studied the benefits from a laboratory efficiency point of view. This is the first large, multicenter study in North America to report on the benefits derived from automation measured in full-time equivalents (FTE), FTE reallocation, productivity, cost per specimen, and cost avoidance. Pre- and post-full automation audits were done at 4 laboratories that have vastly different culture volumes, and results show that regardless of the size of the facility, improved efficiencies can be realized after implementation of full laboratory automation.

## Abstract (precis)

Automation in clinical microbiology is starting to become more commonplace and reportedly offers several advantages over the manual laboratory. Most studies have reported on the rapid turnaround times for culture results, including times for identification of pathogens and their respective antimicrobial susceptibilities, but few have studied the benefits from a laboratory efficiency point of view.

## INTRODUCTION

For the past several decades, clinical laboratories have been asked to do more with less, and diagnostic microbiology has been no exception. We are asked to accurately detect antibiotic-resistant microorganisms, screen patients for hospital-acquired infections, provide more and more rapid results to assure decreased patient length of stay, rapidly test hundreds to thousands of patients daily for severe acute respiratory syndrome coronavirus 2 (SARS-CoV-2), and more. All of this is in the face of decreasing laboratory resources. We are receiving less and less reimbursement for the testing we perform, undergoing laboratory consolidations (often without accompanying resources for the increased workload), receiving unfunded legislative mandates, undergoing budget cuts, and facing significant decreases in workforce, all of which make it very challenging to meet these expectations.

Laboratory automation came to our clinical colleagues in chemistry, hematology, and immunology decades ago and more recently has reached microbiology. There are now two fully automated specimen processing and aerobic culture systems available to microbiology laboratories as follows: Copan’s solution (Murrieta, CA and Brescia, Italy) consisting of the Walk-Away specimen processor (WASP)-DT and the WASPLab full laboratory automation (FLA) system and Becton-Dickinson’s system (Sparks, MD) consisting of the InoqulA and the Kiestra total laboratory automation (TLA). Both offer automated specimen processors, smart incubators, track lines, and digital imaging for the examination of cultures displayed on a computer screen without the need to manually handle the plates. In addition, Copan offers artificial intelligence and interpretive algorithm (AI/IA) software that can segregate positive and negative cultures, provide colony counts, and screen positive from negative results obtained from chromogenic agars. BD is also working toward providing algorithms for culture reading.

Studies have been published on some of the benefits of these systems, and several are listed here as references (1–16). However, to date there has not been a systematic, well-controlled, multicenter study on the total work efficiencies that can be realized once these systems are in place and functional. This is the first report of a such a study utilizing Copan’s full laboratory automation (FLA) system.

## MATERIALS AND METHODS

This study was conducted at 4 facilities of different size, patient mix and acuity, and geographic location. A variety of data was collected 6 months to 1 year before installment of FLA and then again after automation had been implemented (February 2019 to February 2020) based on the laboratory processes in place at that time. Data collected at each site were the same pre- and postanalysis and included full-time equivalent (FTE) number, FTE allocation (based on peak day scheduling and workbench staffing across all shifts), and the total volume of culture specimens received, processed, and worked up. Post-FLA FTE salaries for laboratory assistants and clinical laboratory scientists were provided by the laboratory, or a national salary average approved by participants was used (laboratory assistants, $60,000; clinical laboratory scientists, $90,000). From this data, we were able to determine FTE productivity, labor cost per specimen, and cost avoidance associated with automated culture processing and workup. Specifically, each laboratory had a workflow analysis performed before and after implementation of their FLA, which included review of the daily and peak volumes of specimens processed and the number and scheduling of FTE allocated for culture processing and workup. If turnaround time (TAT) data were available, this was also included. Pre- and post-FLA workflow analysis was performed by a Six Sigma Black Belt professional Copan consultant who spent 2 to 3 days onsite observing laboratory operations before and after FLA at each site.

### Calculations.

1. Labor cost per specimen. Total daily labor cost/total volume of culture specimens processed on peak day.
2. Productivity. Volume of cultures processed or volume of cultures worked up/number FTE required on peak day to complete the work.
3. Total productivity percentage. Post-FLA productivity minus pre-FLA productivity/pre-FLA productivity.
4. FTE needed without FLA. FTE that would be predicted without FLA (adjusting for volume growth; for example, if a laboratory required 1 FTE to complete the work pre-FLA and grew 50% post-FLA, the adjusted FTE needed without automation would equal 1 FTE + 50% or 1.5 FTE).
5. FTE savings or cost avoidance with growth. Pre-FLA FTE subtracted from FTE needed without FLA.
6. Direct savings FLA. Post-FLA FTE subtracted from Pre-FLA FTE.
7. Total FTE savings (direct savings and cost avoidance). Add cost avoidance with growth to direct FTE savings with FLA.
8. Turnaround time (TAT). Time of culture set up subtracted from the time of the final report (derived from the laboratory information system data).

Note that all costs were derived including only specimens for which FLA is used and that FTE was determined by the number of hours spent processing specimens and working up cultures.

### Laboratory demographics, FLA instrumentation, and workflow overview.

Willis-Knighton Laboratory (WKL) services a 5-hospital network and outreach program extending throughout Louisiana and processes approximately 143,000 bacterial cultures annually. The pre-FLA analysis was performed in 2016 (annual volume of bacterial cultures excluding blood cultures, 81,030), and post-FLA analysis was done in 2019 (annual volume of bacterial cultures excluding blood cultures, 118,625). No automation was present at the time of the laboratory’s pre-FLA evaluation, and no algorithms were implemented in this laboratory. The WKL FLA solution consisted of a single line connecting 2 WASP and 3 smart incubators. Pre-FLA, WKL received 70% of culture volume processed off-site and operated a day shift culture reading and workup process only. Post-FLA, WKL processes 100% of cultures on-site and operates 24/7 for culture reading and workup. FLA processes 87% of their total culture volume (Table 1).

**TABLE 1:** Laboratory instrumentation and workflow overview

Condition	Instrumentation or workflow overview for:^*a*^			
	WKL	TC	HRMLP	SHSL
Pre-FLA instrumentation	None	None	None	2 WASP
Post-FLA instrumentation	2 WASP, 1 track line, 3 incubators	1 WASP, 2 incubators	2 WASP, 1 track line, 3 incubators	3 WASP, 2 track lines, 6 incubators
Specimen processing	Pre-FLA, 24/7 with 70% of volume processed off-site; post-FLA, 24/7 with 100% volume processed on-site	Pre-FLA, 24/7; post-FLA, 24/7	Pre-FLA, 24/7; post-FLA, 24/7	Pre-FLA, 24/7; post-FLA, 24/7
Culture workup	Pre-FLA, 08:00–19:00; post-FLA, 24/7	Pre-FLA, workup 07:00–00:30; post-FLA, 05:30-02:30	Pre-FLA, 08:00–17:00; post-FLA, 08:00–23:00 (positive blood cultures: 24/7)	Pre-FLA, 24/7; post-FLA, 24/7
Specimens/collections processed by WASP/FLA (percent of laboratory volume)	Urine, MRSA, ESwab, GBS, body fluid, positive blood culture (87% FLA)	Urine, MRSA, ESwab, respiratory, GAS, body fluid, disc diffusion AST (90% WASP; 81% FLA)	Urine, MRSA, VRE, GAS, CSF, positive blood culture (88% WASP; 80% FLA)	Urine, MRSA, GAS, GBS, body fluid, stool (96% FLA)
Segregation algorithms	No	Yes: urine	Yes: urine, MRSA, VRE, MRSA/VRE biplate	Yes: urine, MRSA
Years between pre- and post-FLA analysis	3	5	8	3

a WLK, Willis-Knighton Laboratory; TC, TriCore Reference Laboratories; HRMLP, Hamilton Regional Medicine Laboratory Program; SHSL, Sutter Health Shared Laboratory; WASP, Walk-Away specimen processor; FLA, full laboratory automation; MRSA, screening culture for MRSA; GBS, screening culture for group B *Streptococcus*; GAS, screening culture for group A *Streptococcus*; AST, antimicrobial susceptibility testing; VRE, screening culture for VRE; ESwab, specimens collected in ESwab devices; CSF, cerebrospinal fluid.

TriCore (TC) Reference Laboratories is New Mexico’s largest medical laboratory serving numerous hospitals, physicians’ offices, and collection centers statewide. TC processes approximately 775,000 bacterial cultures per year. The pre-FLA analysis was performed in 2015 (annual volume of bacterial cultures excluding blood cultures, 318,280), and post-FLA analysis was done in 2018 (annual volume of bacterial cultures excluding blood cultures, 395,295), 4 years after automation was complete and segregation software for urine cultures was fully integrated. No automation was present at the time of the laboratory’s pre-FLA evaluation. TC now processes approximately 90% of their specimens on the single WASP instrument, and urine cultures and methicillin-resistant *Staphylococcus aureus* (MRSA) screens (approximately 81% of their volume) are processed on an FLA line consisting of the WASP instrument and 2 smart incubators. Since FLA, TC was able to extend their hours of culture workup to help improve TAT (Table 1).

Hamilton Regional Medicine Laboratory Program (HRMLP) is a laboratory reference center providing testing services to 11 hospitals and a cancer center within the Hamilton, ON, Canada area. They process approximately 600,000 bacterial cultures annually. Analysis was performed in 2012 before automation (annual volume of bacterial cultures excluding blood cultures, 349,305), and post-FLA analysis was done in 2020 (annual volume of bacterial cultures excluding blood cultures, 571,225), 8 years after FLA had been successfully implemented and 3 years after algorithm software programs were in routine use. HRMLP had no microbiology automation prior to 2013. HRMLP processes 88% of all specimens on their 2 WASP instruments, and 80% of specimens are placed on their FLA line consisting of 2 WASP instruments, 1 track line, and 3 smart incubators. Post-FLA analysis also included the use of urine segregation and MRSA and vancomycin-resistant enterococci (VRE) segregation software. They were able to extend their culture workup hours from 1 to 2 shifts (Table 1).

Sutter Health Shared Laboratory (SHSL), located in northern California, serves 24 hospitals and 26 clinics. The laboratory processes over 600,000 bacterial cultures annually. Analyses were performed in 2016 pre-FLA (annual volume of bacterial cultures excluding blood cultures, 410,625), and again in 2019 post-FLA (annual volume of bacterial cultures excluding blood cultures, 601,620). Prior to FLA, SHSL was using 2 WASP instruments, which increased to 3 WASP instruments, 2 track lines, and 6 smart incubators at their post-FLA evaluation. SHSL post-FLA analysis also included the use of urine segregation and MRSA segregation software. Approximately 96% of all specimens are processed, and cultures worked-up, using FLA (Table 1).

## RESULTS

### Willis-Knighton Laboratory.

Pre-FLA specimens were processed for culture at 3 of its hospitals utilizing 0.3 FTE at each location (determined by the time laboratory assistants spent processing cultures). These cultures were incubated at the processing site until they were transported (approximately 20 min) to WKL for continued incubation and workup. These “off-site” processed cultures represented approximately 70% of their total culture volume. After the implementation of FLA, all specimens were transported to WKL for processing on the WASP, which resulted in an immediate 0.9 FTE savings (Table 2).

**TABLE 2:** Pre- and post-FLA metrics

Lab and metric^*a*^	Pre-FLA					Post-FLA				
	FTE	Total labor cost/day ($)	Specimen vol: peak day	Specimens/FTE (productivity)	Labor cost/specimen ($)	FTE	Total labor cost/day ($)	Specimen vol: peak day	Specimens/FTE (productivity)	Labor cost/specimen ($)
WKL										
Processing	2.9	317.81	222	77	1.43	2	219.18	325	163	0.67
Culture Work up	5	958.90	222	44	4.32	4	767.12	325	81	2.36
Total	7.9	1,276.71	222	28	5.75	6	986.30	325	54	3.03
TC										
Processing	7	1,150.68	872	125	1.32	4	657.52	1,083	271	0.61
Culture Work up	9	2,219.18	872	97	2.54	12	2,794.51	1,083	90	2.58
Total	16	3,369.86	872	55	3.86	16	3,452.03	1,083	68	3.19
HRMLP										
Processing	5	821.92	957	191	0.86	7	1,150.68	1,565	224	0.74
Culture Work up	18	4,438.36	957	53	4.64	17	4,191.78	1,565	92	2.68
Total	23	5,260.27	957	42	5.50	24	5,342.47	1,565	65	3.41
SHSL										
Processing	13	2,315.07	1,125	87	2.06	13	2,315.07	1,648	87	2.06
Culture Work up	17	5,775.34	1,125	66	5.13	23.5	7,720.62	1,648	70	4.68
Total	30	8,090.41	1,125	38	7.19	36.5	10,035.69	1,648	45	6.09

a Some laboratories provided actual FTE salaries while others preferred to use a national average of $90,000/year for a clinical laboratory scientist and $60,000/year for a laboratory assistant.

Prior to FLA implementation, the system processed approximately 222 specimens per day at peak times. This increased to 325 specimens during their post-FLA evaluation, accounting for an increase of 46% in specimen volume. Productivity increased from 77 to 163 specimens processed per FTE and from 44 to 81 cultures worked up per FTE for a combined productivity increase of 93%. Total FTE needed for combined specimen processing and culture workup decreased from 7.9 pre-FLA to 6 post-FLA, resulting in a direct savings of 1.9 FTE. However, when adding in the cost avoidance associated with a 46% volume increase, an additional 3.7 FTE were saved accounting for a total savings of 5.6 FTE or approximately $322,000 annually in labor costs (Table 3). As a result, the labor cost per specimen decreased 47% from $5.75 per specimen to $3.03 per specimen (Table 2). Additionally, improvements were seen in the TAT of results. Before the implementation of FLA at WKL the average urine culture TAT was 30.8 h. This decreased to 23.0 h after FLA implementation, resulting in a 7.8 h more rapid TAT for these results. If WLK had maintained their pre-FLA work schedule (08:00 to 19:00) in the postanalysis period, they would have improved TAT for 73% (5.7 h decrease) for urine cultures. This leaves approximately 27% (2.1 h) of the improved TAT attributable to extending culture workup hours of 24 h/day.

**TABLE 3:** FTE savings with FLA

Lab and metric	Pre-FLA	FLA	Direct savings FLA	FTE needed without FLA	FTE savings cost avoidance with growth	Total FTE savings (direct savings + cost avoidance)	Total FTE cost savings/yr ($)
WKL							
Peak volume	222	325	325	325			
Processing	2.9	2	0.9	4.2	1.3	2.2	89,819.82
Workup	5	4	1	7.3	2.3	3.3	232,387.39
Total	7.9	6	1.9	11.6	3.7	5.6	322,207.21
TC							
Volume	872	1,083	1,083	1,083			
Processing	7	4	3	8.7	1.7	4.7	281,628.44
Workup AST		2	−2			−2.0	(120,000.00)
Workup	9	10	−1	11.2	2.2	1.2	105,997.71
Total	16	16	0	19.9	3.9	3.9	267,626.15
HRMLP							
Volume	957	1565	1565	1565			
Processing	5	7	−2	8.2	3.2	1.2	70,595.61
Workup	18	17	1	29.4	11.4	12.4	1,119,216.30
Total	23	24	−1	37.6	14.6	13.6	1,189,811.91
SHSL							
Volume	1125	1648	1,648	1,648			
Processing	13	13	0	19.0	6.0	6.0	392,831.00
Workup AST		4	−4			−4.0	(400,000.00)
Workup	17	19.5	−2.5	24.9	7.9	5.4	669,985.78
Total	30	36.5	−6.5	43.9	13.9	7.4	662,816.78

### TriCore Reference Laboratories.

Prior to FLA implementation, the system processed approximately 872 specimens per day at peak times. This increased to 1,083 specimens during their post-FLA evaluation, accounting for an increase of 24% in specimen volume. Productivity increased from 125 to 271 specimens processed per FTE, and while cultures worked up per FTE went from 97 to 90, a combined productivity increase of 24% was seen. Total FTE needed for combined specimen processing and culture workup remained the same at 16 for pre-FLA and post-FLA. When adding in the cost avoidance associated with a 24% volume increase with no additional FTE added (cost avoidance of 3.9 FTE), this accounted for approximately $268,000 annually in labor costs (Table 3). As a result, the labor cost per specimen decreased 17% from $3.86 per specimen to $3.19 per specimen (Table 2).

**FIG 1:**
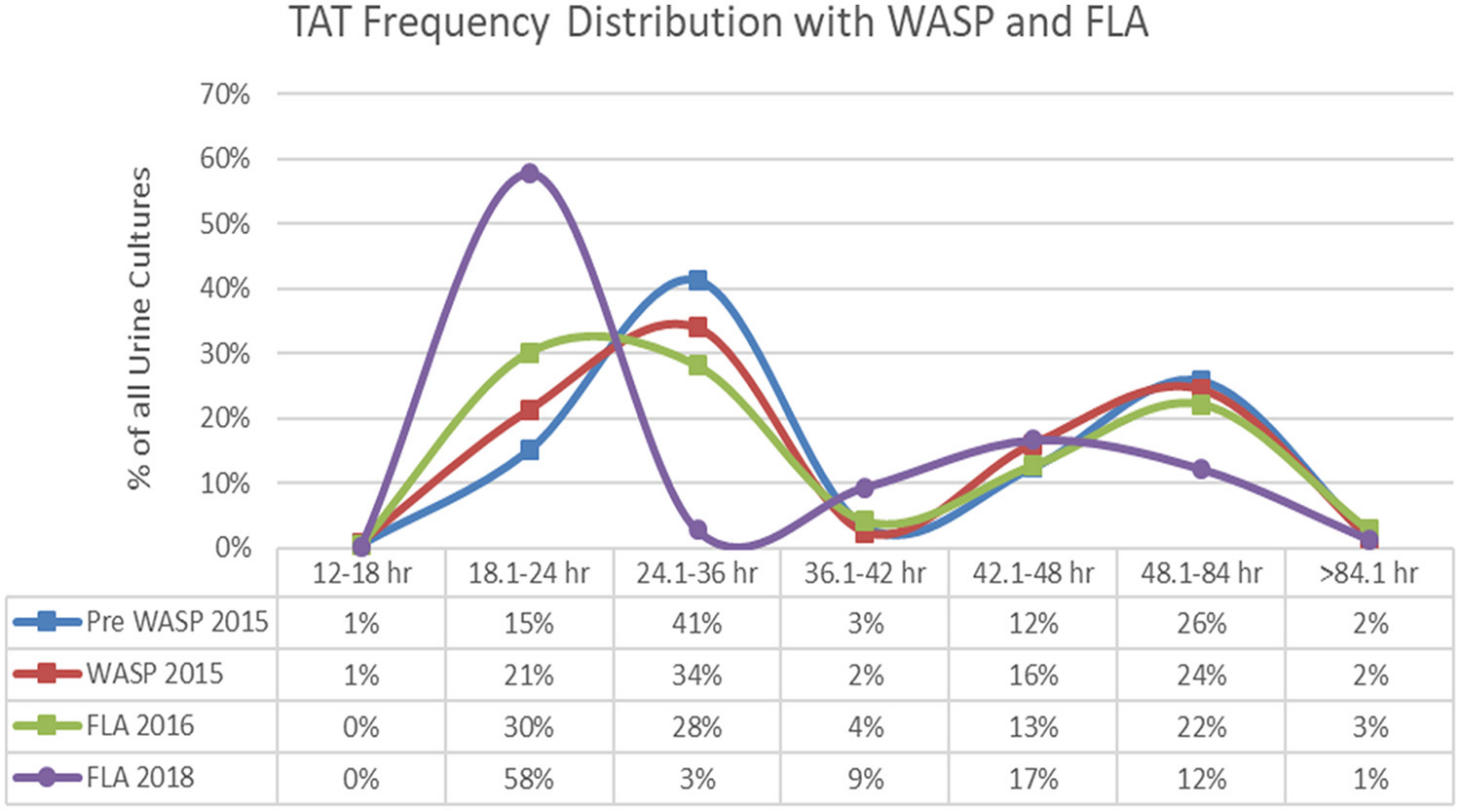
Turnaround times for TC through evaluation period. Pre-WASP 2015, no automation in the clinical laboratory; WASP 2015, initial implementation of the WASP alone; FLA 2016, initial integration of FLA; FLA 2018, implementation of algorithm-assisted FLA.

They were also able to add approximately 3.5 additional hours of culture workup, extending their total time from 17.5 h pre-FLA to 21 h post-FLA. In addition, TC was able to decrease their TAT throughout each step of their automation process. Their median TAT preautomation, post-WASP, post-FLA, and after implementation of reading algorithms was 35 h, 31 h, 29 h, and 21 h, respectively. This median decrease of 14 h was accompanied by a 24% increase in specimen volume. Approximately 11% of all urine cultures were ready for workup between 00:30 and 07:00 pre-FLA when staff were not scheduled. Extending culture reading time by 3.5 h, only 4% of cultures were ready to read when staff were not scheduled. This suggests that up to 7% (11% minus 4%) of TAT improvement could be directly attributed to extending culture reading hours (assuming staff were working up cultures at optimal incubation times as can be achieved through FLA). As stated previously, the median TAT improvement or decrease was 14 h, and as 7% of this was estimated to be due to the extension of culture reading times (14 h × 7% = 0.98 h), then approximately 13 h or 93% of this improvement was associated with FLA alone. Figure 1 shows the TATs at TC Laboratories as they went from no automation, to working with a WASP, to implementation of FLA, and then finally to algorithm-assisted FLA. The percentage of specimens in the first curve (0 to 36 h) for algorithm-assisted FLA and (0 to 42 h) for pre-WASP, WASP, and FLA 2016 when added together for each time frame is as follows: pre-FLA = 60%, WASP = 58%, FLA 2016 = 63%, and FLA 2018 = 61%. Approximately 60% of specimens represent those cultures reported as equivalent to no growth, normal flora, mixed growth, contamination, insignificant growth, etc., basically those that do not require additional workup in the laboratory. As exemplified in the 18.1- to 24-h time frame, with algorithm-assisted FLA, the number of these specimens released is almost 4 times that of no automation, 3 times that of WASP alone, and approximately twice that of FLA without algorithms. The remaining part of the graph, or the second curve (>36.1 h for algorithm-assisted FLA and >42 h for the 3 other time frames), shows that pre-WASP = 40%, WASP = 42%, FLA 2016 = 37%, and FLA 2018 = 39% for cultures requiring additional work up in the laboratory, such as subcultures, identifications, susceptibility testing, etc. In the category of cultures that take >36 h but <42.1 h to generate a final report, there is an increase in the percentage of specimens reported with algorithm-assisted FLA (9% compared to 3% with no automation, 2% WASP, and 4% FLA 2016). This is due to the shift of the curve to the left for algorithm-assisted FLA with earlier reporting of cultures that require workup, e.g., the second curve begins earlier with algorithm-assisted FLA. Lastly, there is a stepwise decrease in the percentage of cultures taking >48 h to a final report with the addition of each part of automation.

### Hamilton Regional Medicine Laboratory Program.

Prior to FLA implementation, the system processed approximately 957 specimens per day at peak times. This increased to 1,565 specimens during their post-FLA evaluation, accounting for an increase of 64% in specimen volume. Productivity increased from 191 to 224 specimens processed per FTE and from 53 to 92 cultures worked up per FTE for a combined productivity increase of 55%. Total FTE needed for combined specimen processing and culture workup increased from 23 pre-FLA to 24 post-FLA; however, when adding in the cost avoidance associated with a 64% volume increase, an additional 13.6 FTE were saved accounting for just under $1,200,000 annually in labor costs (Table 3). As a result, the labor cost per specimen decreased 38% from $5.50 per specimen to $3.41 per specimen (Table 2). FLA with culture algorithms significantly contributed to the observed efficiencies for culture workup activities and negated the need for additional FTE despite an increased workload of 64% over the 8-year period.

### Sutter Health Shared Laboratory.

Prior to FLA implementation, the system processed approximately 1,125 specimens per day at peak times. This increased to 1,648 specimens during their post-FLA evaluation, accounting for an increase of 46% in specimen volume. Productivity remained constant for specimens processed per FTE at 87 for both study periods but increased from 66 to 70 cultures worked up per FTE for a combined productivity increase of 18%. Total FTE needed for combined specimen processing and culture workup went from 30 pre-FLA to 36.5 post-FLA, resulting in an increase of 6.5 FTE; however, when adding in the cost avoidance associated with a 46% volume increase, 7.4 FTE were saved or approximately $663,000 annually in labor costs (Table 3). As a result, the labor cost per specimen decreased 15% from $7.19 per specimen to $6.09 per specimen (Table 2).

## DISCUSSION

These laboratory experiences demonstrate that FLA can be applied to a variety of laboratories large and small, reference laboratories to hospital-based laboratories, and from those processing and working up a few hundred cultures per day to those that process and read thousands each day. The laboratories in this study all benefited from savings in FTE regardless of their size, regardless of the increase in specimen volume seen during the pre- and poststudy periods, and regardless of the years when these analyses were performed. WKL saved 1.9 FTE directly and a total of 5.6 FTE with cost avoidance based on increased specimen volume during the study period. These improvements were a function of processing a wide variety of specimens with FLA, including tissues that were manually ground and placed on the WASP for inoculation, streaking, and incubation. Processing steps that were eliminated with automation included selection of plates, transport of plates to the setup area, labeling of plates, planting/streaking of plates and broths, and transporting plates to incubator. For the offsite laboratories, it also saved FTE by eliminating the packing of plates for transport, planting/streaking of plates and broths, etc. Even though SHSL added 6.5 FTE during the study period, with their increase in culture volume, they avoided hiring an additional 7.4 FTE. Likewise, with the efficiencies gained by FLA, TC, having the same FTE pre- and postanalysis, avoided hiring 3.9 FTE equaling a total of over $250,000 in FTE savings. HRMLP required the addition of only 1 FTE post-FLA even while seeing a 64% increase in specimen volume between the study periods. In addition, they experienced a decrease in labor of over $2.00 in cost per specimen and a productivity increase of 55%.

Increases in productivity ranged from 18% to 93% with the implementation of FLA. The FTE savings and increases in productivity seen by these laboratories were accompanied by a decrease in cost per specimen ranging from 15% to 47%, resulting in annual labor savings of between ∼$268,000 to ∼$1.2 million. FLA also allowed those laboratories that were not already processing and working up cultures 24/7 to do so or to add additional hours of culture workup more easily than if they were still a manual laboratory. To emphasize this point, the TAT data at TC, where automation allowed an expansion of culture reading times, showed a median reduction of ∼14 h for culture results to be reported. Even with cultures that need additional incubation time, identification, and/or susceptibility testing performed, an advantage is still seen in this area for FLA. This is shown in the data from TC (Fig. 1), where the second curve representing the TAT for algorithm-assisted reporting has the greatest number (9%) of positive cultures reported between 36.1 and 42 h. One can also see a decreasing volume of all cultures that are reported at greater than 48 h through each step of automation with 28%, 26%, 25%, and 13% of cultures being reported for pre-FLA, WASP, FLA 2016, and FLA 2018, respectively. This may be explained in that FLA in general, and algorithm-assisted reading specifically, allows more efficient release of no growth and insignificant cultures, so the work on more significant cultures can begin sooner. Algorithm assistance may equate to less time spent on managing these types of cultures, leaving more time for assessment of complex cultures.

The fact that microbiologists are retiring at an accelerated rate, that fewer personnel are entering the field of laboratory medicine, and that reimbursements are declining, laboratories are left with little choice but to find ways to innovate and produce quality results with fewer and fewer personnel. Laboratory automation can offer some solutions to these challenges. This study shows that FTE savings can be realized not only in the upfront specimen processing area but in culture workup as well. FLA affords these savings on the bench by allowing technologists to visually review images from both positive and negative cultures in batch mode for rapid release of results and faster workup of specimens continually throughout the day, evening, and overnight shifts. The laboratory having the greatest volume growth and FTE savings (HRMLP) utilizes segregation algorithms for 80% of their cultures; the reduction in time to screen their large volume of negative cultures contributes to these savings. In addition, continuous incubation afforded by laboratory automation shortens the time to culture results (8–10). FLA allows cultures to be reviewed, worked up, and reported when they are optimally ready rather than when staff is scheduled to be in the laboratory. It is true that simply switching to a 24/7 processing and culture workup schedule without FLA will allow for specimens to be read more appropriately at their optimal incubation times, but as previously stated, the display of batched images afforded by algorithms allows for even more savings in time spent reviewing cultures as well as allowing earlier result reporting times.

In summary, this is the first large scale study in North America to look at the efficiencies and cost-savings that can be realized when implementing full laboratory automation in the bacteriology culture laboratory. Overall, such laboratory automation can achieve significant benefits relating to FTE, productivity, specimen cost, TAT, etc. These benefits will allow microbiology laboratories to continue to provide high-quality results in the midst of continuing declining resources and support. Incorporation of tools such as artificial intelligence with interpretative culture algorithms together with future improvements in the automated release of negative routine and chromogenic culture results will continue to provide the microbiology community with much needed efficiencies. Additional innovations, e.g., complicated customized ruled-based resulting, as well as rapid, inexpensive antimicrobial susceptibility setup and interpretation will be available soon. These and future developments will continue to have significant applicability in microbiology laboratories.
